# Reduction of Rice Noodle Rehydration Time by High-Temperature Short-Time Treatment

**DOI:** 10.3390/foods14071079

**Published:** 2025-03-21

**Authors:** Xudong Yan, Hong Xiao, Jiangping Ye, Shunjing Luo, Chengmei Liu

**Affiliations:** 1 State Key Laboratory of Food Science and Resources, Nanchang University, Nanchang 330047, China; 2 International Institute of Food Innovation, Nanchang University, Nanchang 330200, China

**Keywords:** rice noodle, rehydration, porous structure, multiscale structure, starch

## Abstract

Rapid rehydration is a critical challenge in the production of dry rice noodles. This study investigated the impact of high-temperature short-time treatments (HTSTTs) at temperatures of 120 °C, 130 °C, and 140 °C for 80 s on the rice noodle rehydration time and the underlying mechanisms. HTSTT led to a reduction in the relative crystallinity and molecular weight of starch, along with the disruption of its supramolecular structure. Moreover, significant alterations were observed in the pore properties after HTSTT, characterized by a notable increase in total pore volume and average pore size. The enhanced porosity and disrupted starch multiscale structure resulted in shortened cooking times for the rice noodles. This reduction in cooking time mitigated gel disruption during cooking, thereby reducing amylose leaching and preserving a more intact gas cell wall structure. Consequently, HTSTT markedly enhanced the overall quality of the rice noodles. For instance, noodles treated at 140 °C for 80 s exhibited a 26.55% decrease in cooking time, a 30.37% reduction in cooking loss, a 37.62% increase in hardness, and a 13.24% increase in resilience compared to the control group. In summary, HTSTT emerges as a feasible method for reducing the cooking time of rice noodles.

## 1. Introduction

Rice noodle is a traditional staple in many countries, particularly in China and Southeast Asia. Based on moisture content, rice noodles can be classified into dry, semi-dry, and wet varieties [1]. The dry rice noodle dominates the market due to its long shelf life and ease of storage and transportation. However, it requires rehydration before consumption to restore desirable qualities, such as hardness, chewiness, and elasticity [2]. The extended rehydration time remains a limitation to the development of the dry rice noodle industry. Various strategies have been employed to enhance the rehydration properties of rice noodles, including raw material selection [3], raw material modification [4,5,6], process parameter optimization [7,8], and the incorporation of additives [2,9,10]. Given the growing consumer demand for “clean-label” foods, natural, additive-free rice noodles with rapid rehydration may be more attractive to consumers.

Drying is a critical step in the production of dry rice noodles, and the porous structure formed during this process significantly influences their rehydration performance. However, different drying methods result in varying degrees of pore formation. For instance, deep frying and vacuum freeze drying can create extensive pore structures that significantly reduce rehydration time. However, the high-fat content in deep frying and the expensive equipment required for vacuum freeze drying limit their widespread use [11,12]. In contrast, hot-air drying has been widely adopted in the rice noodle industry due to its lower cost. However, due to the slower heat and mass transfer rates in hot-air drying, rice noodles are more likely to experience structural shrinkage and collapse, leading to a denser texture. As a result, hot-air drying creates fewer pores, resulting in a significantly longer rehydration time compared to other drying methods [13]. To address this challenge, optimizing the process parameters of hot-air drying is crucial for improving the structural properties of rice noodles. However, existing studies have primarily focused on enhancing drying efficiency [14] and improving edible qualities (e.g., cooking loss and texture characteristics) [15], with less emphasis placed on rehydration time. We hypothesize that employing a high-temperature short-time treatment (HTSTT) could effectively shorten the rehydration time by rapidly evaporating moisture, thereby forming numerous micropores within the noodle and partially disrupting the starch multiscale structure.

This study aimed to examine the effect of HTSTT on the rice noodles’ quality, with a specific emphasis on its impact on rehydration time. Furthermore, the effects of HTSTT on the porous structure of rice noodles and the multiscale structure of starch were systematically analyzed to elucidate the mechanism behind the decreased rehydration time. The findings not only provide a theoretical basis for addressing the rehydration challenges of dry rice noodles but also propose a novel technological approach to foster innovation and development within the convenience-food industry.

## 2. Materials and Methods

### 2.1. Materials

Early indica rice (amylose content: 22.93%) was obtained from Guangzhou Jinhongda Grain and Oil Co., Ltd. (Guangzhou, China). Corn starch (amylose content: 24.20%) was sourced from Jilin COFCO Biochemical Energy Sales Co., Ltd. (Changchun, China). Protease (EC 3.4.21.62) was purchased from Megazyme Co., Ltd. (Wicklow, Ireland). Chromatography-grade dimethyl sulfoxide (DMSO) was supplied by Shanghai Anpu Experimental Technology Co., Ltd. (Shanghai, China). Unless otherwise stated, all reagents were of analytical grade.

### 2.2. Preparation of Rice Noodles

Rice noodles were prepared according to our previously reported method [16]. The overall process of preparing rice noodles is shown below:

Soaking rice → milling → mixing corn starch → removing excess water → extrusion → retrogradation → saturated steam heat moisture treatment → drying

Retrogradation and previous procedures were identical to those previously reported [16]. At the end of the retrogradation, the rice noodles underwent saturated steam-heat moist treatment for 10 min. The rice noodles were then subjected to high-temperature short-time treatment (HTSTT) using a tunnel oven (FMDR-200, Hunan Fumach Food Engineering Technology Co., Ltd., Changsha, China). According to the principle of “minimizing the rehydration time of rice noodles while preventing macroscopic expansion and browning”, the final treatment temperatures were set at 120 °C, 130 °C, and 140 °C, with a duration of 80 s. The rice noodles were ultimately dried at 45 °C with 60% relative humidity for 3 h to achieve the desired dryness. Some of the dried noodles were reserved for quality assessment, while the remaining portion was ground and sieved through a 100-mesh screen for analyzing the multiscale starch structure.

### 2.3. Measurement of Physical Properties of Rice Noodles

The color of rice noodles was determined by a colorimeter (CM-23d Spectrophotometer, Konica Minolta Holdings, Inc., Tokyo, Japan), including lightness (L*), redness (a*), and yellowness (b*) values. The whiteness index (WI) of rice noodles was calculated according to Equation (1), and the overall color difference (ΔE) between the treated samples and the control group was calculated according to Equation (2) [17]:

(1) $$WI=100-\sqrt{{\left(100-L^{*}\right)}^{2}+{\left(a^{*}\right)}^{2}+{\left(b^{*}\right)}^{2}}$$

(2) $$∆E=\sqrt{{\left({∆L}^{*}\right)}^{2}+{\left({∆a}^{*}\right)}^{2}+{\left({∆b}^{*}\right)}^{2}}$$

Ten rice noodles, each 10 cm in length, were selected, and their diameters were measured using vernier calipers.

The bulk density of rice noodles was calculated using Equation (3), following the method described by Janve et al. [18].

(3) $$Bulk\;Density\left(\frac{g}{{cm}^{3}}\right)=\frac{4\times m}{\pi \times D^{2}\times L}$$

where m represents the weight of the rice noodles, L denotes their length, and D refers to their diameter. Ten randomly selected rice noodles were measured.

### 2.4. Measurement of Cooking Properties

The optimal cooking time was determined, with slight modifications, according to a previous report [19]. The procedure involved immersing 5 g of rice noodles in 200 mL of deionized water at 90 °C. Nearing rehydration completion, rice noodles were removed every 10 s, pressed between glass plates, and visually examined. The optimal cooking time was determined when the white core completely disappeared. To evaluate rehydration properties, 5 g of rice noodles (m_0_) was immersed in 200 mL of 90 °C deionized water for the optimal cooking time. After cooking, the noodles were rinsed with 200 mL of cold deionized water, and excess moisture was removed with filter paper before weighing (m_1_). The combined liquids were dried at 105 °C to a constant weight (m_2_). Rehydration rate and cooking loss were then calculated using Equations (4) and (5).

(4) $$Rehyration\;rate\left(\%\right)=\frac{m_{1}-m_{0}}{m_{0}}\times 100\%$$

(5) $$Cooking\;loss\left(\%\right)=\frac{m_{2}}{m_{0}}\times 100\%$$

### 2.5. Determination of Textural Properties

The evaluation of rice-noodle textural properties was conducted based on the method outlined by Yan et al. [16]. The procedure consisted of first using filter paper to gently remove surface moisture from two cooked rice noodles, which were then placed into a texture analyzer for assessment. Texture profile analysis (TPA) was carried out with a texture analyzer (TA.XT plus, Stable Micro System, Surrey, UK) equipped with an HDP/PFS probe. Test conditions were consistent with those previously reported [16].

### 2.6. Determination of Microstructure

The freeze-dried cooked rice noodles were sliced into 2 mm thick cross-sectional pieces and secured onto a cylindrical sample stage using double-sided conductive tape. Following gold sputter coating, the samples’ microstructure was examined with scanning electron microscopy (Regulus 8100, Hitachi High-Tech Co., Ltd., Tokyo, Japan). The average area of the gas cell and the thickness of the gas cell wall were calculated by Image J software (version 1.8.0).

### 2.7. Sensory Evaluation

The sensory evaluation of rice noodles was carried out by 10 trained sensory evaluators according to the previous report with minor modifications [18]. Cooked rice noodles were placed in white bowls, each labeled with a randomly assigned three-digit number. Sensory evaluators were provided with water and were required to wash and swallow between samples. Each quality attribute (appearance, aroma, taste, texture, and overall acceptability) of the rice noodle was scored using a 9-point hedonic scale (9 = extremely like, 5 = neither like nor dislike, and 1 = extremely dislike).

### 2.8. Measurement of the Crystalline Structure

The crystal structure of the dried rice noodles was analyzed using an X-ray diffractometer (D8 ADVANCE, Bruker Corporation, Billerica, USA), following the method outlined by Luo et al. [20]. The spectra were recorded at a scanning speed of 1.2°/min, within a diffraction angle range from 5° to 35° (2θ). Jade 9.0 software (Materials Data Company, CA, USA) was utilized to evaluate the relative crystallinity of the different crystal types.

### 2.9. Determination of Supramolecular Structure

The samples’ water content was adjusted to 60% and equilibrated at room temperature for 24 h. Afterward, spectral data were collected using a small-angle X-ray scattering (SAXS; Xeuss 2.0, Xenocs, Grenoble, France), covering the range from 0.007 to 0.40 Å^−1^. Data within the 0.02–0.25 Å^−1^ range were fitted using Equation (6) [21].

(6) $$I\left(q\right)=I_{L}\left(0\right)\frac{1}{{\left[1+\frac{D^{f}+1}{3}\left(q^{2}\xi^{2}\right)\right]}^{\frac{D^{*}}{2}}}+I_{G}\left(0\right)\exp\left(-\frac{q^{2}R^{2}}{3}\right)+B$$

In this equation, I_L_(0) represents the Lorentz parameter, I_G_(0) denotes the Guinier scale factor, ξ refers to the correlation length, and R signifies the gyration radius.

Finally, the fractal structure of rice noodle powder was determined by fitting the data in the low-q region using the power law equation (Equation (7)) for calculation.

(7) $$I\left(q\right)\sim q^{\alpha }$$

The exponent (α) represents the slope of the SAXS curve when plotted on a double logarithmic scale [22].

### 2.10. Determination of Molecular Weight

Starch was extracted and purified according to the method described by Yan et al. [16]. The molecular weight of the samples was analyzed using size exclusion chromatography (SEC) (1260 Infinity II, Agilent Technologies, Santa Clara, CA, USA) [23]. Briefly, 1.5 mL of DMSO solution containing 0.5% (*w*/*w*) LiBr was added to a centrifuge tube with 4–6 mg of the isolated starch. The mixture was then incubated in a shaker at 350 rpm and 80 °C for 24 h. After incubation, the mixture was centrifuged at 15,000 rpm for 10 min, and the supernatant was collected for further analysis. Then, 100 μL of supernatant was injected into the SEC system for analysis, and the eluent consisted of 99.5% DMSO and 0.05% LiBr. The column oven was set at 60 °C, and the elution rate was maintained at 0.3 mL/min. The molecular weight of the starch was expressed as both the weight-average molecular weight (Mw) and the number-average molecular weight (Mn).

### 2.11. Determination of Pore Structure

The porous structure of rice noodles was thoroughly analyzed using a specific surface area analyzer (Autosorb IQ, Anton-Paar, Graz, Austria). Initially, rice noodles (approximately 1 cm in length) were precisely weighed and placed into pre-weighed glass test tubes. These samples were then degassed at 60 °C for 10 h to eliminate water and volatile substances, including gases trapped within the pores. Following the degassing process, nitrogen was introduced into the sample pores. The mass of the degassed rice noodles was recorded, and nitrogen adsorption–desorption isothermal experiments were conducted at a temperature of 77.3 K. To determine the pore size distribution, desorption isotherms were analyzed using the Barrett–Joyner–Halenda (BJH) model, with relative nitrogen pressures (P/P0) ranging from 0.05 to 0.30 [8].

### 2.12. Statistical Analysis

Each test was performed at least three times, and the results were expressed as the mean ± standard deviation. Statistical analyses were conducted using SPSS software (version 24.0), with group differences assessed through one-way analysis of variance (ANOVA), followed by Tukey’s post hoc test. A significance level of *p* < 0.05 was considered statistically meaningful.

## 3. Results and Discussion

### 3.1. Physical Properties of Rice Noodles

**Figure 1:**
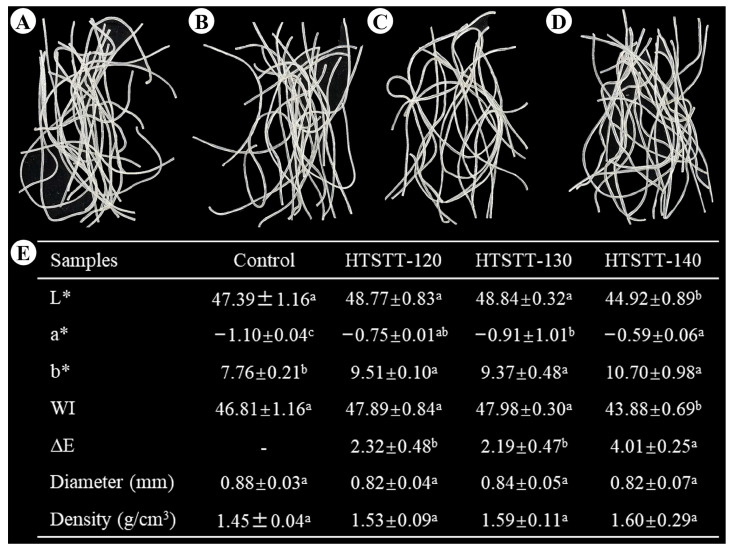
Appearance (**A**–**D**) and physical properties (**E**) of rice noodles: (**A**) control, (**B**) HTSTT-120, (**C**) HTSTT-130, and (**D**) HTSTT-140. L*, lightness; a*, redness; b*, yellowness; WI, whiteness index. All data were expressed as mean ± standard deviation (n ≥ 3). Different superscript lowercase letters in the same row indicate significant differences among the samples. The control group represented rice noodles that were not subjected to high-temperature short-term treatment (HTSTT). HTSTT-X meant that the HTSTT temperature was X °C.

The appearance and physical properties of rice noodles are shown in Figure 1. There were no significant changes in the diameter (0.82–0.84 mm) and density (1.53–1.60 g/cm^3^) of the HTSTT rice noodles compared to the control group (0.88 mm for diameter; 1.45 g/cm^3^ for density). These results indicated that HTSTT did not induce puffing in rice noodles. These results were desirable, as preliminary experiments revealed that macroscopic puffing of rice noodles led to a high breakage rate and cooking losses.

Color is an important indicator for evaluating dry rice noodles, and it affects consumer choice and acceptance. Figure 1 shows that the color of rice noodles was affected by HTSTT to some extent. HTSTT did not significantly alter the L* and WI values of rice noodles compared to the control group, except for HTSTT-140. However, The HTSTT group showed higher a* and b* values compared to the control group. This indicated that HTSTT rice noodles were darker in color and higher in redness and yellowness than the control group. This was attributed mainly to the high temperatures that promoted the Maillard reaction [24]. Similarly, rice noodles that have undergone heat moisture treatment displayed a darker color [25]. Consumers can perceive color differences when the ΔE value exceeds 2, while ΔE > 5 is considered a significant difference [26]. Although color differences existed between the HTSTT rice noodles and the control group, ΔE < 5 indicated that these differences were not significant.

### 3.2. Cooking Properties of Rice Noodles

**Figure 2:**
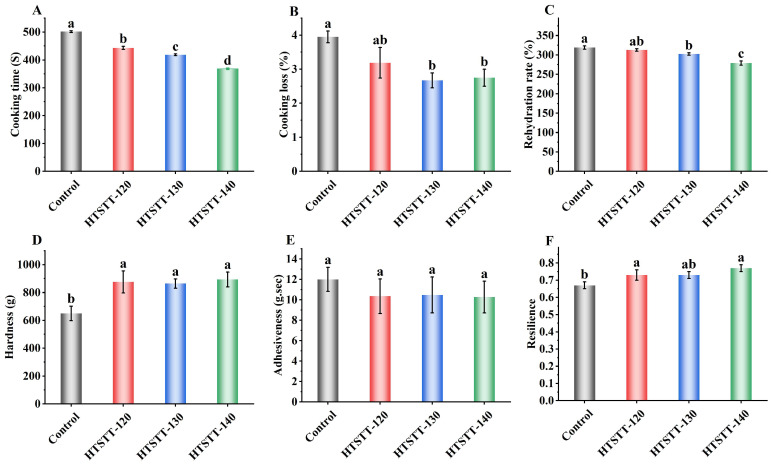
The cooking time (**A**), cooking losses (**B**), rehydration rate (**C**), hardness (**D**), adhesiveness (**E**), and resilience (**F**) of rice noodles. Different lowercase letters on the bars indicate significant differences in the samples.

**Figure 3:**
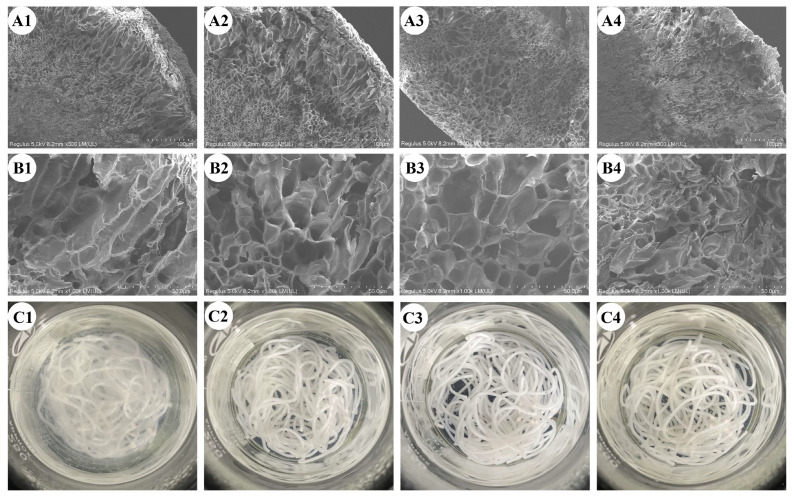
Microstructure of cooked rice noodles magnified 300 × (**A1**–**A4**) and 1000 × (**B1**–**B4**), and turbidity of cooking water: (**A1**–**C1**) control, (**A2**–**C2**) HTSTT-120, (**A3**–**C3**) HTSTT-130, and (**A4**–**C4**) HTSTT-140.

The cooking properties are critical indicators of dried rice noodle quality. As shown in Figure 2A, HTSTT significantly reduced the optimal cooking time and rehydration rate of rice noodles. Specifically, the cooking time significantly decreased from 502 s to a range of 368–443 s, while the rehydration rate declined from 318.89% to 279.13–312.76%. The reduction in the optimal cooking time may be attributed to changes in the multiscale structure of starch [2,27] and modifications in the pore structure of rice noodles [8], which will be discussed in detail in subsequent sections. The decrease in rehydration rate was likely due to the shortened cooking time, which restricted the amount of water that could penetrate the rice noodles. After HTSTT, the water for cooking rice noodles became clearer (Figure 3), indicating a reduction in cooking losses. To confirm this, cooking loss was measured (Figure 2B). The results demonstrated a significant reduction (*p* < 0.05) in the cooking loss of rice noodles following HTSTT. Specifically, the cooking loss was 3.95% in the control group, whereas it decreased to 3.67% for HTSTT-120, 3.19% for HTSTT-130, and 2.75% for HTSTT-140. Cooking loss primarily resulted from the structural breakdown of rice noodles and the dissolution of gelatinized starch [28]. The observed reduction in cooking loss may be attributed to changes in the multiscale and pore structures of the rice noodles, which enhanced water penetration into the interior and reduced structural damage during cooking. Similarly, Xiang et al. [15] reported that starch noodles dried under high-temperature and high-humidity conditions, followed by high-temperature hot-air drying, exhibited lower cooking losses compared to those processed with conventional hot-air drying. In summary, HTSTT improved the cooking properties of rice noodles, as evidenced by a shorter cooking time and lower cooking loss.

### 3.3. Microstructure of Cooked Rice Noodles

The micromorphological characteristics of cooked rice noodles are shown in Figure 3. The cross-section revealed a porous honeycomb structure, with these pores resulting from moisture sublimation during the freeze-drying stage [29]. Similarly, this porous structure was observed in cooked rice [29] and rice noodles [30]. Notably, Figure 3A showed uneven gas cell distribution, with larger gas cells near the edges and smaller gas cells at the center. During cooking, rice noodles transitioned from a dry gel to a hydrogel, and the water permeated from the outside to the inside regions, leading to a gradient in moisture distribution. The higher moisture content at the outer edges may induce excessive swelling, potentially damaging the overall integrity of the gel structure [31]. To further investigate the impact of HTSTT on the microstructure of rice noodles, the edge area was magnified 1000 times (Figure 3B). There was no significant change in the micropore area for HTSTT-120 (64.52 µm^2^) and HTSTT-130 (68.61 µm^2^) compared to the control group (65.02 µm^2^), whereas HTSTT-140 (39.65 µm^2^) exhibited a significant decrease (Table 1). Moreover, HTSTT rice noodles displayed a thicker gas cell wall. Specifically, the gas cell wall thickness was 0.73 µm for the control group, 0.87 µm for HTSTT-120, 1.09 µm for HTSTT-130, and 0.76 µm for HTSTT-140. This reduction in gas cell size and the increase in gas cell wall thickness was likely linked to the shortened cooking time, which reduced the damage to the gel structure of rice noodles during the cooking process.

### 3.4. Texture Properties of Cooked Rice Noodles

The textural characteristics of cooked rice noodles are critical determinants of consumer acceptance [32]. It was reported that rice noodles that had a hard texture, were chewy and elastic, and were non-sticky were preferred by the market, and these characteristics have become important indicators of the quality of commercially available rice noodles [1,33]. As shown in Figure 2E,F, rice noodles subjected to HTSTT exhibited a notable increase in hardness and elasticity. Meanwhile, adhesiveness showed a slight reduction, though the change was not statistically significant. Specifically, in the HTSTT-140 group, compared to the control, hardness increased from 650 g to 821 g, resilience rose from 0.70 to 0.77, and adhesiveness decreased from 12.00 g⋅s to 10.27 g⋅s. The increased hardness was primarily attributed to a decrease in gas cell size, leading to a higher density of gas cell walls per unit area, along with a slight thickening of the gas cell walls in cooked rice noodles after HTSTT (Figure 3B and Table 1). It was reported that the gas cell wall played a crucial role in determining the mechanical strength of products with porous structures, with thicker gas cell walls significantly enhancing hardness [34]. Additionally, a positive relationship was observed between the hardness of cooked rice noodles and their amylose content [35]. The reduced cooking losses in rice noodles treated with HTSTT suggested that a smaller amount of amylose was leached during cooking. This resulted in higher amylose retention, which consequently enhanced the hardness of the noodles. Resilience is a key parameter that reflects the rubbery characteristics of rice noodles. HTSTT shortened the optimal cooking time, which helped preserve the structural integrity of the gel network, thereby enhancing resilience [25]. The slight decrease in adhesiveness was likely due to reduced cooking losses, leading to less starch adhering to the noodle surface [36,37]. In summary, HTSTT significantly improved the quality of rice noodles, as evidenced by a shorter cooking time, reduced cooking losses, and increased hardness.

### 3.5. Sensory Evaluation

**Figure 4:**
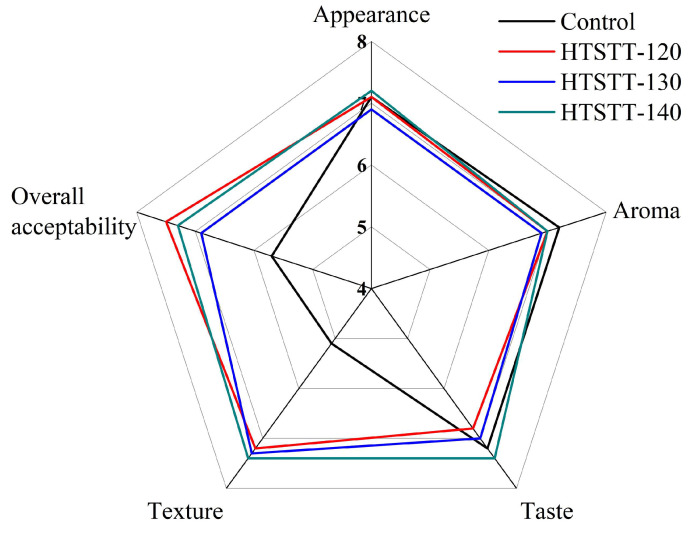
The sensory evaluation of rice noodles.

Figure 4 presents the sensory evaluation results of HTSTT rice noodles at various temperatures. HTSTT improved the texture and overall acceptability scores of rice noodles, which may be attributed to HTSTT improving the hardness and resilience of rice noodles. There was no significant difference in the appearance score of all rice noodles, and this result was attributed to the fact that HTSTT did not cause noticeable expansion or color changes. In addition, HTSTT did not significantly affect the aroma and taste scores of rice noodles, suggesting that it did not induce notable undesirable flavors. Overall, the sensory evaluation results further confirmed the feasibility of HTSTT to enhance rice noodles.

### 3.6. Changes in the Multiscale Structure of Starch in Rice Noodles

As the primary component of rice noodles, the multilevel structural properties of starch are likely intrinsically linked to its edible quality. Building on this assumption, this study systematically analyzed the multiscale structural changes in starch in rice noodles to investigate the mechanism by which HTSTT improves its edible quality.

#### 3.6.1. Crystal Structures

**Figure 5:**
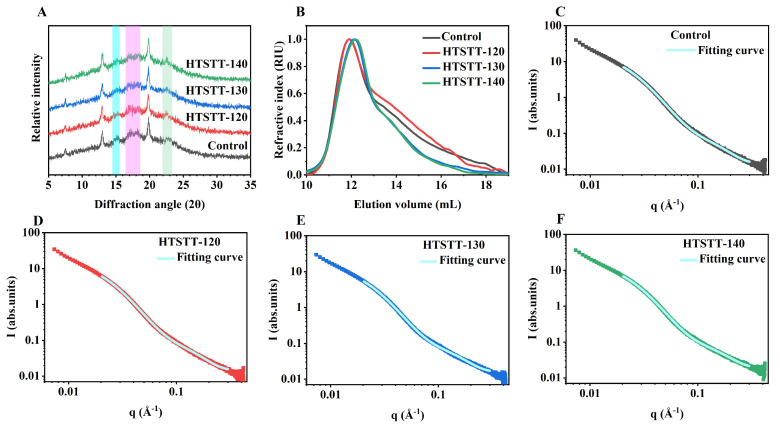
X-ray diffraction pattern (**A**), molecular weight distribution (**B**), and small-angle scattering fitting curve (**C**–**F**) of rice noodles.

The X-ray diffraction patterns of rice noodles are shown in Figure 5A. HTSTT had no significant effect on the characteristic diffraction peaks, with all rice noodle samples displaying distinct peaks at 15°, 17°, 18°, and 23°, indicative of the A-type crystalline structure. The development of A-type crystals was primarily linked to the retrogradation of starch occurring under conditions of high temperatures (50 °C) and low moisture levels (20–25%) [38,39], as these diffraction peaks were absent in samples directly obtained from the extrusion mold. This finding was consistent with our previous results [16,40]. Additionally, distinct diffraction peaks were detected at 7°, 13°, and 20° across all samples, corresponding to V-type crystal structures. The formation of V-type crystals may result from amylose interacting with small amounts of free fatty acids (mainly palmitic, oleic, and linoleic acids) in rice to form an amylose–lipid complex [41,42]. The presence of starch–lipid complexes contributed to lowering starch digestibility and stabilizing postprandial blood glucose levels [43,44]. Similar results were reported for extrusion-treated kudzu powder [45] and rice starch [23].

HTSTT led to a significant decrease in the crystallinity of the A-type diffraction peak in rice noodles. Specifically, the A-type crystallinity of the control group was 21.33%, whereas that of the HTSTT-120, HTSTT-130, and HTSTT-140 groups decreased to 11.76%, 10.21%, and 9.17%, respectively (Table 2). This phenomenon was primarily due to the gelatinization and thermal degradation of starch at high temperatures, leading to the disruption of its crystalline structure [46,47]. It has been reported that amorphous regions demonstrate higher hydrophilicity than crystalline regions [48]. Therefore, the decrease in crystallinity may facilitate faster water penetration into the interior of the rice noodles. Similarly, extracellular polysaccharides produced through *Weissella confusa* fermentation reduced noodle crystallinity, thereby shortening the rehydration time [27].

#### 3.6.2. Supramolecular Structure

The small-angle X-ray scattering (SAXS) technique was employed to characterize the supramolecular structure of rice noodles. As shown in Figure 5C–F, the SAXS spectra did not reveal distinct characteristic peaks within the scattering vector (q) around 0.7 nm^−1^, implying that the sample lacked a characteristic starch-layered arrangement. In contrast, a broad “shoulder peak” around 0.3 nm^−1^ was observed, pointing to the formation of a partially ordered structural arrangement in the rice noodles, which was consistent with previous findings in retrograded starches [49,50]. To further analyze the starch structure, we followed the method proposed by Rosciardi et al. [21], where the SAXS curves were fitted to extract two key parameters: gyration radius (R) and correlation length (ξ). The gyration radius (R) reflects the size of the tie-points formed by amylopectin crystals. After HTSTT, the R-value decreased from 12.31 nm to 10.99–11.98 nm, likely due to the dissociation of part of the double helix structure in starch molecules under high temperature, leading to smaller tie-points, consistent with the observed reduction in crystallinity. The correlation length (ξ) represents the average distance between polymer chains and the mesh size of the gel network. In the rice noodle system, amylose forms an elastic gel network through molecular entanglements, which serve as the backbone structure of the noodles. As a result, in this study, the ξ value is indicative of the mesh size within the three-dimensional gel network formed by amylose. It was observed that the ξ value of the rice noodles increased significantly following the HTSTT, suggesting an enlargement of the mesh size within the gel network. Larger mesh sizes in the gel network may accelerate the entry of water into the interior of the rice noodles, thereby helping to mitigate damage to the gel structure during cooking. As a result, HTSTT led to a reduction in cooking losses and an increase in the hardness and resilience of the rice noodles.

The fractal dimension of the system can be assessed using the exponent, α, in the power law equation (Equation (7)). When α falls within the range from 1 to 3, it signifies a mass fractal structure, where higher α values correspond to increased structural density. Conversely, when α lies between 3 and 4, the system exhibits surface fractal characteristics. As presented in Table 2, the α values of all rice noodle samples remained within the 1-to-3 range, confirming their mass fractal nature. This observation aligned with previous research findings, which indicated that gelatinized starches predominantly exhibited mass fractal behavior [51,52]. Compared to the control group (α = 1.65), the samples treated with HTSTT displayed a slight decrease in their mass fractal dimension, with a notable reduction in the HTSTT-140 sample. This suggested that HTSTT disrupted the ordered arrangement of the starch double helix (i.e., the crystalline lamellar structure), leading to a loosening of the overall structure [53]. This looser structure may provide a more efficient pathway for water penetration, which helped to explain the observed significant reduction in the cooking time of rice noodles following HTSTT. Similarly, previous studies have reported that a decrease in the fractal dimension of starch in rice noodles facilitated faster rehydration [2,28].

#### 3.6.3. Molecular Weight

The molecular-weight results of starch in rice noodles are presented in Figure 5B and Table 2. After HTSTT, the elution volume of starch showed a significant shift to the right, indicating starch-molecule degradation. As detailed in Table 2, HTSTT notably reduced the Mn and Mw of starch in rice noodles. For instance, the Mn and Mw of the control starch were 5.05 × 10^6^ g/mol and 15.78 × 10^6^ g/mol, respectively, whereas those for HTSTT-140 decreased to 2.63 × 10^6^ g/mol and 8.45 × 10^6^ g/mol, respectively. This reduction in molecular weight can be attributed to the high temperature disrupting the molecular structure of starch and causing the breaking of glycosidic bonds [54]. Similar reductions in molecular weight have been observed in starch subjected to heat treatments such as baking [55] and frying [54].

### 3.7. Changes in the Pore Structure of Rice Noodles

**Figure 6:**
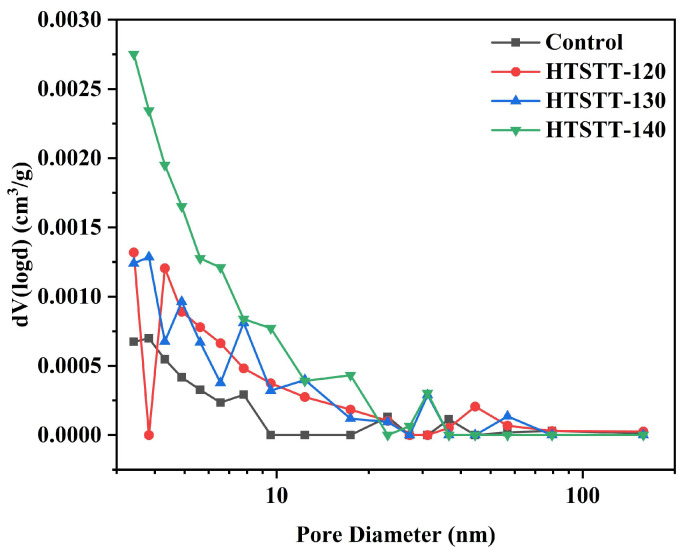
Pore size distribution of rice noodles.

The pore size distribution curve of rice noodles is shown in Figure 6. After HTSTT, the pore volume distribution curve shifted upward, indicating an increase in the number of pores within the rice noodles. The pore volume and average pore size of rice noodles were determined using the BJH method, and the results are presented in Table 2. HTSTT significantly increased the pore volume and average pore diameter. The pore volume of the control group was 3.05 × 10⁻^3^ cm^3^/g, and the average pore diameter was 3.06 nm. For HTSTT-120, HTSTT-130, and HTSTT-140, the pore volumes increased to 4.96 × 10⁻^3^ cm^3^/g, 6.70 × 10⁻^3^ cm^3^/g, and 11.19 × 10⁻^3^ cm^3^/g, respectively, while the average pore diameters were 3.17 nm, 3.41 nm, and 3.69 nm. The increase in pore volume and size was attributed to the rapid evaporation of water at high temperatures, leading to the formation of numerous pores within the rice noodles. It was well-established that a porous structure facilitated water migration, enhancing the rehydration of dry starch products, and larger pore sizes were particularly favorable for rehydration [8,56]. In conclusion, HTSTT improved the pore structure of rice noodles, thereby contributing to their rapid rehydration.

### 3.8. Possible Mechanisms

**Figure 7:**
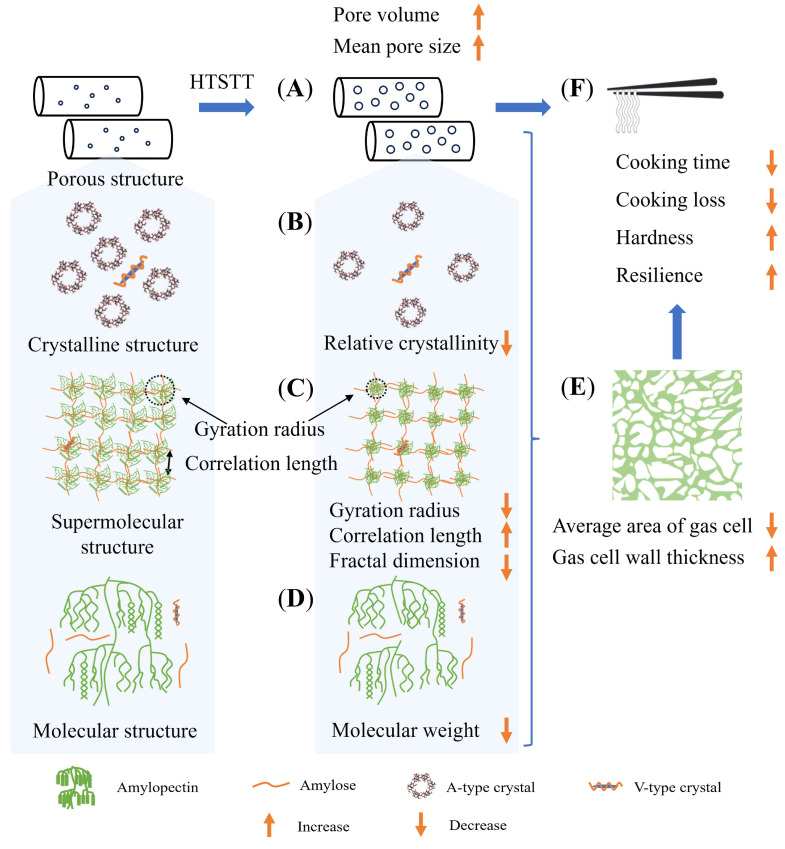
Diagram of possible mechanisms for accelerated rehydration of rice noodles by high-temperature short-time treatment. (**A**): Porous structure of dried rice noodles; (**B**): Crystalline structure; (**C**): Supramolecular structure; (**D**): Molecular structure; (**E**): Microstructure of cooked rice noodles; (**F**): Quality evaluation of rice noodles.

After HTSTT, rice noodles exhibited a shortened cooking time, lower cooking losses, and enhanced firmness and resilience, without macroscopic puffing and discoloration. Figure 7 illustrates the potential mechanisms behind these improvements. The reduction in cooking time was attributed to two primary factors: (1) the rapid evaporation of water during HTSTT led to an increase in pore volume and average pore size (Figure 7A), facilitating faster water absorption into the dried rice noodles; and (2) HTSTT induced partial damage to the multiscale structure of starch, including a decrease in crystallinity (Figure 7B), an enlargement of the gel network mesh size and loosening of the overall structure **(**Figure 7C), and the breaking of starch molecular chains (Figure 7D). As discussed previously, these structural changes in starch likely contributed to the accelerated rehydration. The shortened cooking time reduced the damage to the rice noodle gel during cooking, decreasing the leaching of amylose and preserving the integrity of the gas cell wall (Figure 7E), which enhanced the mechanical strength of the rice noodle. Consequently, the results showed that the quality of rice noodles was significantly improved by HTSTT, with shorter cooking time, lower cooking loss, and higher hardness and resilience, as well as higher sensory evaluation scores (Figure 7F).

## 4. Conclusions

HTSTT significantly reduced the cooking time and improved the overall quality of rice noodles. Specifically, HTSTT enhanced porosity and disrupted the multiscale structure of starch, leading to a shorter cooking time. This reduction in cooking time decreased damage to the rice noodle gel network during cooking, thereby reducing the leaching of amylose and preserving the integrity of the gas cell wall. This, in turn, contributed to the enhanced gel strength of the rice noodles. Consequently, rice noodles subjected to HTSTT showed reduced cooking time; reduced cooking losses; and increased hardness, resilience, and sensory evaluation scores. This study provides a feasible method for the production of rice noodles with rapid rehydration properties. However, the cooking time of rice noodles in this study remained above 6 min. In the future, high-temperature short-time treatment combined with other approaches (e.g., food additives) could be explored to further reduce the rehydration time of rice noodles.

## Figures and Tables

**Table 1:** The average area of the micropores and the thickness of the gas cell walls.

Samples	Control	HTSTT-120	HTSTT-130	HTSTT-140
Average area (μm^2^)	65.02 ± 0.74 ^a^	64.52 ± 1.95 ^a^	68.61 ± 1.36 ^a^	39.65 ± 1.81 ^b^
Thickness (μm)	0.73 ± 0.03 ^c^	0.87 ± 0.03 ^b^	1.09 ± 0.04 ^a^	0.76 ± 0.05 ^c^

All data were expressed as mean ± standard deviation (n = 3). Different superscript letters in the same row indicated significant differences between samples. The control group was rice noodles that were not subjected to high-temperature short-time treatment (HTSTT). HTSTT-X indicated that the HTSTT temperature was X °C.

**Table 2:** Multiscale structural parameters of starch in rice noodles.

Samples	Control	HTSTT-120	HTSTT-130	HTSTT-140
Relative crystallinity (%)	28.29 ± 0.11 ^a^	18.66 ± 0.61 ^b^	17.34 ± 0.18 ^c^	16.88 ± 0.69 ^c^
A-type crystallinity (%)	21.33 ± 0.17 ^a^	11.76 ± 0.15 ^b^	10.21 ± 0.10 ^bc^	9.17 ± 1.39 ^c^
V-type crystallinity (%)	6.96 ± 0.27 ^a^	6.90 ± 0.46 ^a^	7.13 ± 0.27 ^a^	7.71 ± 0.70 ^a^
ξ (nm)	10.97 ± 0.68 ^b^	11.37 ± 0.76 ^ab^	11.98 ± 0.39 ^ab^	12.88 ± 0.44 ^a^
R (nm)	12.31 ± 0.40 ^a^	11.41 ± 0.51 ^ab^	11.98 ± 0.39 ^ab^	10.99 ± 0.47 ^b^
α	1.65 ± 0.01 ^a^	1.63 ± 0.01 ^ab^	1.62 ± 0.01 ^b^	1.58 ± 0.01 ^c^
Mn (×10^6^ g/mol)	5.05 ± 0.08 ^a^	4.75 ± 0.10 ^b^	4.06 ± 0.06 ^c^	2.63 ± 0.08 ^d^
Mw (×10^6^ g/mol)	15.78 ± 0.08 ^a^	14.47 ± 0.12 ^b^	12.16 ± 0.12 ^c^	8.45 ± 0.15 ^d^
pore volume (×10^−3^ cm^3^/g)	3.05 ± 0.01 ^d^	4.96 ± 0.87 ^c^	6.70 ± 0.98 ^b^	11.19 ± 0.04 ^a^
Average pore diameter (nm)	3.06 ± 0.00 ^b^	3.17 ± 0.21 ^b^	3.41 ± 0.00 ^ab^	3.69 ± 0.24 ^a^

All data were expressed as mean ± standard deviation (n = 3). Different superscript letters in the same row indicated significant differences between samples. ξ, correlation length; R, gyration radius; α, fractal dimension; Mn, number-average molecular weight; Mw, weight-average molecular weight.

## Data Availability

The original contributions presented in this study are included in the article. Further inquiries can be directed to the corresponding authors.

## Abbreviations

The following abbreviations are used in this manuscript:

HTSTT	High-temperature short-time treatment
Mn	number-average molecular weight
Mw	weight-average molecular weight
DMSO	dimethyl sulfoxide
SEC	size exclusion chromatography
